# Multimodality cellular and molecular imaging of concomitant tumour enhancement in a syngeneic mouse model of breast cancer metastasis

**DOI:** 10.1038/s41598-018-27208-4

**Published:** 2018-06-12

**Authors:** Katie M. Parkins, Veronica P. Dubois, Amanda M. Hamilton, Ashley V. Makela, John A. Ronald, Paula J. Foster

**Affiliations:** 1 Robarts Research Institute, The University of Western Ontario, London, Ontario, Canada; 2 The Department of Medical Biophysics, The University of Western Ontario, London, Ontario Canada; 3 Lawson Health Research Institute, London, Ontario Canada

## Abstract

The mechanisms that influence metastatic growth rates are poorly understood. One mechanism of interest known as concomitant tumour resistance (CTR) can be defined as the inhibition of metastasis by existing tumour mass. Conversely, the presence of a primary tumour has also been shown to increase metastatic outgrowth, termed concomitant tumour enhancement (CTE). The majority of studies evaluating CTR/CTE in preclinical models have relied on endpoint histological evaluation of tumour burden. The goal of this research was to use conventional magnetic resonance imaging (MRI), cellular MRI, and bioluminescence imaging to study the impact of a primary tumour on the development of brain metastases in a syngeneic mouse model. Here, we report that the presence of a 4T1 primary tumour significantly enhances total brain tumour burden in Balb/C mice. Using *in vivo* BLI/MRI we could determine this was not related to differences in initial arrest or clearance of viable cells in the brain, which suggests that the presence of a primary tumour can increase the proliferative growth of brain metastases in this model. The continued application of our longitudinal cellular and molecular imaging tools will yield a better understanding of the mechanism(s) by which this physiological inhibition (CTR) and/or enhancement (CTE) occurs.

## Introduction

Breast cancer is the second leading cause of cancer related deaths in North America with the majority of deaths due to metastasis, the dissemination of cancer cells from the primary tumour to other parts of the body^1^. One of the most common, as well as most fatal sites of metastatic growth for breast cancer patients is the brain, with the incidence of brain metastasis increasing and prognosis remaining poor^2^. Improved knowledge regarding how quickly cancer cells disseminate from the primary tumour and the rate of secondary metastases development, as well as the mechanisms that control proliferation rate, are key to developing new therapies to prevent or halt metastatic growth and prevent cancer mortality.

The mechanisms that influence metastatic growth rates are poorly understood. One mechanism of interest is concomitant tumour resistance (CTR), which describes the ability of the primary tumour to restrict the growth of distant metastases^3,4^. The relevance of CTR has been shown by numerous observations of the removal of a primary tumour being followed by an abrupt acceleration of residual metastatic disease. CTR has been observed in both breast cancer patients^5^ and animal models of breast cancer^6^. It has also been observed in patients with other solid tumour types^7,8^. Conversely, it has been shown that the presence of a primary tumour can likewise increase metastatic outgrowth, a phenomenon coined concomitant tumor enhancement (CTE). However, in the clinic, very few examples of CTE have been reported with most of them being related to suspected regressions of hepatic and/ or pulmonary metastases following nephrectomy for renal cell carcinoma^9–12^. While imaging has been used to describe CTR/CTE effects in patients^13^, the majority of studies evaluating CTR/CTE in preclinical models have relied on endpoint histological evaluation of tumour burden^14,15^. The application of cellular and molecular imaging tools will allow the non-invasive and longitudinal visualization of metastatic progression to study CTR/CTE effects *in vivo*, which will ultimately yield better evaluation of putative molecular mechanism(s) by which this physiological inhibition and/or enhancement occurs. In turn, this may lead to new anti-metastatic therapeutics aimed at these mechanisms and their non-invasive evaluation.

Previously, we applied cellular MRI techniques to study the impact of a primary tumour on metastatic outgrowth in an immune deficient mouse model of experimental breast cancer metastasis to the brain and demonstrated clear CTR effects in the brain^16^. Cellular MRI involves pre-labeling cultured cancer cells with superparamagnetic iron oxide nanoparticles prior to transplantation into mice. This labeling allows transplanted cells to be tracked over time with iron-sensitive MRI techniques. Single cell imaging of cancer cells arresting in the brain at the time of injection and of non-dividing, iron-retaining cancer cells over time is achievable with this technique^17^. However, limitations of iron oxide based cellular MRI are that there is limited ability to differentiate between dead and viable cells, that it is possible for iron particles to be transferred to bystander cells such as macrophages upon cell death, and that the iron particles are diluted during cell division leading to loss of cell detection in proliferative cells. These limitations can be overcome using reporter gene based cell tracking tools, since a stably expressed reporter gene is passed on to daughter cells and will not be expressed in bystander cells. By engineering cells to express a luciferase reporter, bioluminescence imaging (BLI) can provide a direct readout of cell viability in dividing cell populations. Overall, as recently described^18^, combining our highly sensitive cellular MRI tools and BLI yields complementary information on the fate of metastatic cancer cells in preclinical models. As others have pointed to a role of the immune system in CTR/CTE and differences in primary tumour effects have been seen across models of the same cancer type^8^, the purpose of this study was to apply our multimodality imaging tools to study whether CTR/CTE effects are present in the syngeneic 4T1 immune competent mouse model of breast cancer metastasis.

## Materials and Methods

### Cell Labelling and Transduction Procedure

The 4T1-BR5 cells were received from Dr. Patricia Steeg’s lab and engineered to stably co-express red-shifted *Luciola Italica* luciferase (Red-FLuc) and green fluorescent protein (GFP) using a commercial lentiviral vector (RediFect Red-FLuc-GFP; PerkinElmer, USA). Cells were transduced and FACS sorted based on GFP expression using a FACSAria III flow cytometric cell sorter (BD Biosciences). The resultant 4T1BR5-Red-FLuc/GFP cells were maintained in DMEM containing 10% FBS and 1% antibiotics, at 37 °C and 5% CO_2_. For iron labeling, 2 × 10^6^ 4T1BR5-Red-FLuc/GFP cells were plated, and 24 hours later were incubated with 25 μg/mL of micron-sized superparamagnetic iron oxide (MPIO) beads for an additional 24 hours (0.9 μm in diameter, 63% magnetite, conjugated with Flash Red; Bangs Laboratory, Fishers, IN, USA). Cells were washed three times with Hanks balanced salt solution (HBSS), collected and thoroughly washed three more times with HBSS to wash off residual unincorporated MPIO before *in vitro* evaluation or injection into animals.

### *In Vitro* Studies

All *in vitro* results are from three independent experiments with three replicates of each condition. To evaluate the relationship between cell number and BLI signal, 1 × 10^3^, 5 × 10^3^, 1 × 10^4^, 1.5 × 10^5^, and 5 × 10^5^ 4T1BR5-Red-FLuc/GFP cells were seeded in each well of 24-well plates. Twenty-four hours later, 5 μL of D-luciferin (30 mg/mL; Syd Labs, Inc., MA, USA) was added to the growth medium and BLI images were collected 5 minutes later using a hybrid optical/X-ray scanner (IVIS Lumina XRMS *In Vivo* Imaging System, PerkinElmer). BLI signal was measured with region-of-interest (ROI) analysis using LivingImage Software (Perkin Elmer). An ROI was drawn around each well to measure the average radiance (photons/second/mm^2^/steradian), and the mean average radiance across replicates was determined for each independent experiment.

To evaluate if MPIO labeling influenced BLI signal, 1.25 × 10^5^ 4T1BR5-Red-FLuc/GFP cells were seeded in each well of a 24-well plate. Twenty-four hours later, 25 μg/mL of MPIO was added for half of the wells. After an additional twenty-four-hour incubation period, the media for all wells was replaced with fresh media without iron, 5 μL of D-luciferin (30 mg/mL) was added for BLI, and BLI signal per well was analyzed as above.

Vybrant MTT proliferation assays were used to evaluate the effects of genetic engineering on cell proliferation. 6.25 × 10^3^ 4T1BR5-Red-FLuc/GFP or naïve 4T1BR5 cells were seeded in each well of 96 well plates and cells were evaluated 24 and 48 hours later. Twenty microliters of MTT solution was added to each well and absorbance at 450 nm was measured using a microplate spectrophotometer (Fluoroskan Ascent FL, ThermoLabSystems). Cell labeling efficiency was assessed by Perl’s Prussian blue (PPB) staining, as previously described^19^.

### Animal Model

**Figure 1:**
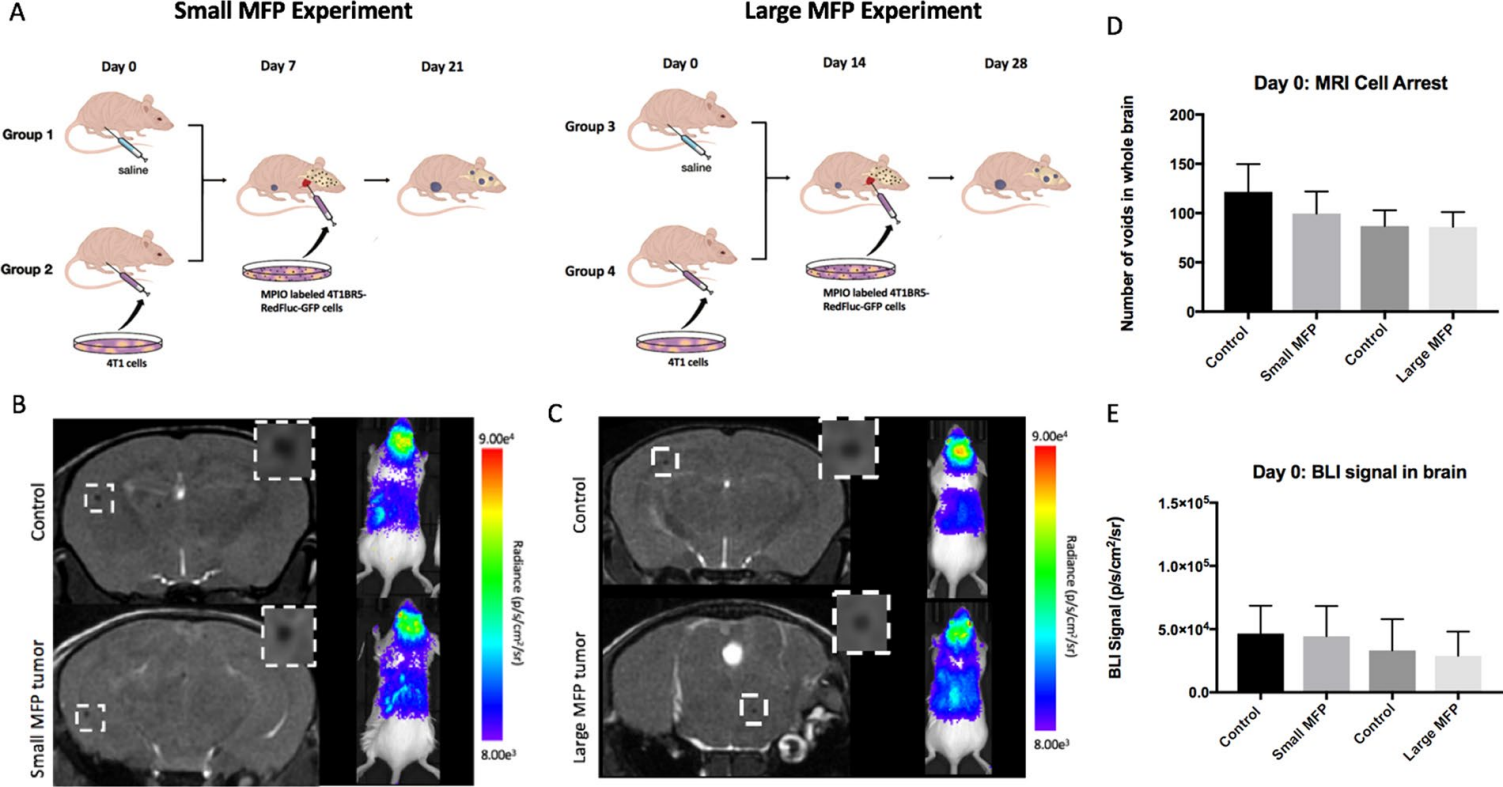
(**A**) Experimental design of animal model used for Small and Large MFP experiments (**B**) On day 0, the number of discrete signal voids, representing iron-labeled cells, that arrested in the brain on day 0 as well as brain BLI signal was not significantly different between mice with and without a small MFP tumour. (**C**) There were also no significant differences in cell arrest for mice with large MFP tumours. (**D**,**E**) All four groups of mice were not significantly different from each other in MRI cell arrest at day 0 as well as BLI signal in the brain at day 0. Data is presented as mean +/− SD.

Figure 1A illustrates the experimental mouse model used in this study. Animals were cared for in accordance with the standards of the Canadian Council on Animal Care, and under an approved protocol of the University of Western Ontario’s Council on Animal Care (2014–026). Six to eight-week-old female BALB/c mice (n = 32) were obtained from Charles River Laboratories (Willington, MA, USA). Mice received a lower right mammary fat pad (MFP) injection of either vehicle (HBSS; Control mice; n = 16) or 300,000 unlabeled 4T1 cells (MFP mice; n = 16). MFP tumour growth was evaluated by measurement with calipers in two perpendicular dimensions, and the tumour volume was estimated using the following formula volume = 0.52 (width)^2^(length), for approximating the volume (mm^3^) of an ellipsoid^20,21^. MFP tumours grew for either seven days (Small MFP) or 14 days (Large MFP) prior to all mice receiving an intracardiac injection of 2 × 10^4^ MPIO-labeled 4T1BR5-Red-FLuc/GFP cells in 0.1 mL of HBSS. Injections were performed under image guidance using a Vevo 2100 ultrasound system (VisualSonics Inc.).

### BLI Procedure

BLI was performed on days 0, 7 and 14 for all mice after intracardiac injection using a hybrid optical/X-ray scanner (IVIS Lumina XRMS *In Vivo* Imaging System, PerkinElmer). Mice were anesthetized with isoflurane (2% in 100% oxygen) using a nose cone attached to an activated carbon charcoal filter for passive scavenging. Anesthetized mice received a 150 μL intraperitoneal injection of D-luciferin (30 mg/mL) and BLI images were captured for up to 35 minutes. On day 0, approximately one hour following intracardiac injection, whole body BLI was used to screen mice for successful intracardiac injection on day 0 and to measure brain BLI signal. Only mice with brain BLI signal proceeded to MRI (i.e., only mice with successful intracardiac injections).

### MRI Procedure

MRI was performed on a 3 T GE clinical MR scanner (General Electric) using custom-built gradient and solenoidal mouse brain radiofrequency coils^22,23^. Mice were anesthetized with isoflurane (2% in 100% oxygen) and images were obtained using a 3D balanced steady state free precession (bSSFP) imaging sequence [Fast Imaging Employing Steady State Acquisition (FIESTA) on the GE system], which has been previously optimized for iron detection^24^. Small MFP and control mice were imaged on days 0, 7, and 14 and large MFP and control mice on days 0 and 14 after intracardiac injection. The scan parameters for day 0 were: repetition time (TR) = 8 ms; echo time (TE) = 4 ms; bandwidth (BW) = 41.7 kHz; flip angle (FA) = 35 degrees; averages (NEX) = 2; phase cycles = 4; matrix = 150 × 150; field-of-view (FOV) = 1.5; resolution: 100 × 100 × 200 μm; and scan time = 19.25 minutes. For days 7 and 14, a longer scan time was required for tumour detection using the following imaging parameters: TR = 10 ms; TE = 5 ms; BW = 12.5 kHz; FA = 35 degrees; NEX = 2; phase cycles = 8; matrix = 150 × 150; FOV = 1.5; resolution = 100 × 100 × 200 μm; scan time = 36.76 minutes. In addition, large MFP mice and corresponding control mice (n = 16), a 3D bSSFP imaging sequence was used to obtain high resolution MR images of the whole mouse body on day 9 using a solenoidal whole-body radiofrequency coil. Sequence parameters were as follows: TR = 6.3 ms; TE = 3.1 ms; BW = 31 kHz; FA = 35 degrees; NEX = 2; phase cycles = 8; matrix = 300 × 150; FOV = 60 × 30 mm; resolution = 200 × 200 × 200 μm^3^; and scan time = 22 minutes. One large MFP mouse and one control mouse also had whole body MR scans at endpoint (day 14) to allow us to match MR and BLI detectable metastases to whole-mouse cryo-fluorescence images as described below.

### Image Analysis

Brain BLI signal was measured with region-of-interest (ROI) analysis using LivingImage Software (Perkin Elmer). An ROI was drawn around the brain, the average radiance (photons/second/mm^2^/sr) was measured, and the peak value over the 35-minute imaging session was used for each mouse at each time point. Whole body BLI signal was measured the same way as listed above with the ROI drawn around the whole mouse body. MRI images were analyzed using OsiriX software (Pixmeo, SARL, Bernex, Switzerland). For days 0 and 7 images, total brain signal void number, representing iron labeled cancer cells, was determined by manually counting voids in every MR slice. For day 14 images, brain metastases were manually traced in every bSSFP image slice for each mouse and 3D tumour volumes were reconstructed using the OsiriX volume algorithm.

### Cryo-Fluorescence Imaging

After endpoint MR imaging (day 14), one large MFP and one control mouse were sacrificed by isoflurane overdose and then flash frozen in OCT freezing medium by liquid nitrogen immersion. The entire mouse was sectioned and optically imaged every 50-μm using a cryo-fluorescence imager (CryoViz^TM^; Bioinvision, Inc., Cleveland, OH). Block-face images were collected with an in-plane resolution of 10.5 × 10.5 μm^2^. Brightfield and fluorescent images were acquired, stitched together and visualized using proprietary software (Bioinvision, Inc).

### Histology

At endpoint, the majority of mice (n = 26) were sacrificed by isoflurane overdose and perfused with 4% paraformaldehyde via the left ventricle. Mouse brains were removed and cryopreserved in ascending concentrations of sucrose (10, 20, and 30% w/v) for 24 hours each. Brains were immersed in optimal cutting temperature (OCT) compound, oriented in a sectioning plane parallel to that of MRI, and frozen using liquid nitrogen. Contiguous 10-μm frozen sections were collected and select sections were stained with hematoxylin and eosin (H&E). Stained sections were imaged using an Invitrogen EVOS FL Auto Cell Imaging System and histological images were manually matched to MR slices using anatomical landmarks such as the ventricles and MR-visible tumours. Fluorescence images of 4T1BR5-Red-FLuc/GFP cancer cells were also collected on the same microscope and matched to MR slices. Spleens were collected and weighed. Contiguous 10-μm paraffin embedded sections were collected, select sections were stained with H&E, and imaged using a Zeiss 510 laser scanning confocal microscope.

### Statistics

A power analysis was performed using G*Power software to determine the appropriate sample size for this study. All statistics were calculated using GraphPad Prism 4. A Shapiro-Wilk normality test found that some of our *in vitro* data was not normally distributed and thus, a non-parametric test (Mann-Whitney) was performed on proliferation experiments. Pearson’s rank correlation was used to determine a relationship between cell number and BLI signal. Student’s two-tailed unpaired t test was used to compare the other *in vitro* experiments as well as between animal groups. A nominal p-value less than 0.05 was considered statistically significant.

### Data availability

All data generated or analyzed during this study are included in this published article (and its Supplementary Information files).

## Results

### *In Vitro* Studies

4T1-BR5 cells were 85.4% transduced with a lentiviral vector and sorted to stably co-express Red-FLuc/GFP (Suppl. 1A,B). The resultant 4T1BR5-Red-FLuc/GFP cells were efficiently (>90%) labeled with MPIO prior to intracardiac injection (Suppl. 1C). There was no significant difference in BLI signal detected in 4T1BR5-Red-FLuc/GFP cells that were labeled with MPIO (2.14 × 10^7^ ± 1.37 × 10^6^ p/s/mm^2^/sr) compared to those that were not labeled (2.48 × 10^7^ ± 1.80 × 10^6^ p/s/mm^2^/sr) (Suppl. 1D). Furthermore, a significant positive correlation was detected between the number of 4T1BR5-Red-FLuc/GFP cells and BLI signal (R^2^ = 0.98, *p* < *0.01*; Suppl. 2A). There were no differences in cellular proliferation detected between naïve 4T1-BR5 and 4T1BR5-Red-FLuc/GFP cells (Suppl. 2B). 4T1-BR5-Red-Fluc/GFP cells also showed no significant change in Red-Fluc expression over multiple passages in culture (Suppl. 2C).

### *In Vivo* Studies

We first looked at imaging data from the day of intracardiac injection (Day 0) shown in Fig. 1. Iron labeled cells were visualized in MR images as discrete signal voids distributed throughout the mouse brain (Fig. 1B,C). Across all 32 mice with a successful intracardiac injection, an average of 98 ± 5 discrete signal voids per mouse were quantified throughout the brains in MR images. The number of discrete signal voids on day 0 was not significantly different between mice with (100 ± 7 voids) and without (121 ± 10 voids) a small primary MFP tumour (Fig. 1D). Similarly, the number of discrete signal voids on day 0 was not significantly different between mice with (86 ± 5 voids) and without (87 ± 6 voids) a large primary MFP tumour (Fig. 1D). BLI signal was also detected in the brain of these thirty-two mice on day 0 (Fig. 1B,C). We found no significant difference in brain BLI signal between mice with (4.4 × 10^4^ ± 8.5 × 10^3^ p/s/mm^2^/sr) and without (4.7 × 10^4^ ± 7.8 × 10^3^ p/s/mm^2^/sr) a small primary MFP tumour, nor with (2.9 × 10^4^ ± 6.8 × 10^3^ p/s/mm^2^/sr) or without (3.3 × 10^4^ ± 8.7 × 10^3^ p/s/mm^2^/sr) a large primary MFP tumour (Fig. 1E).

All MFP tumors were manually assessed with caliper measurements throughout the study. We found a significant difference in Small MFP tumour volume between days 0 (time of intracardiac injection; 138.8 ± 12.11 mm^3^) and 14 (endpoint; 666.4 ± 20.89 mm^3^; p < 0.001). Similarly, we found a significant difference in Large MFP tumour volume between days 0 (308.4 ± 9.14 mm^3^) and 14 (817.9 ± 15.12 mm^3^).

**Figure 2:**
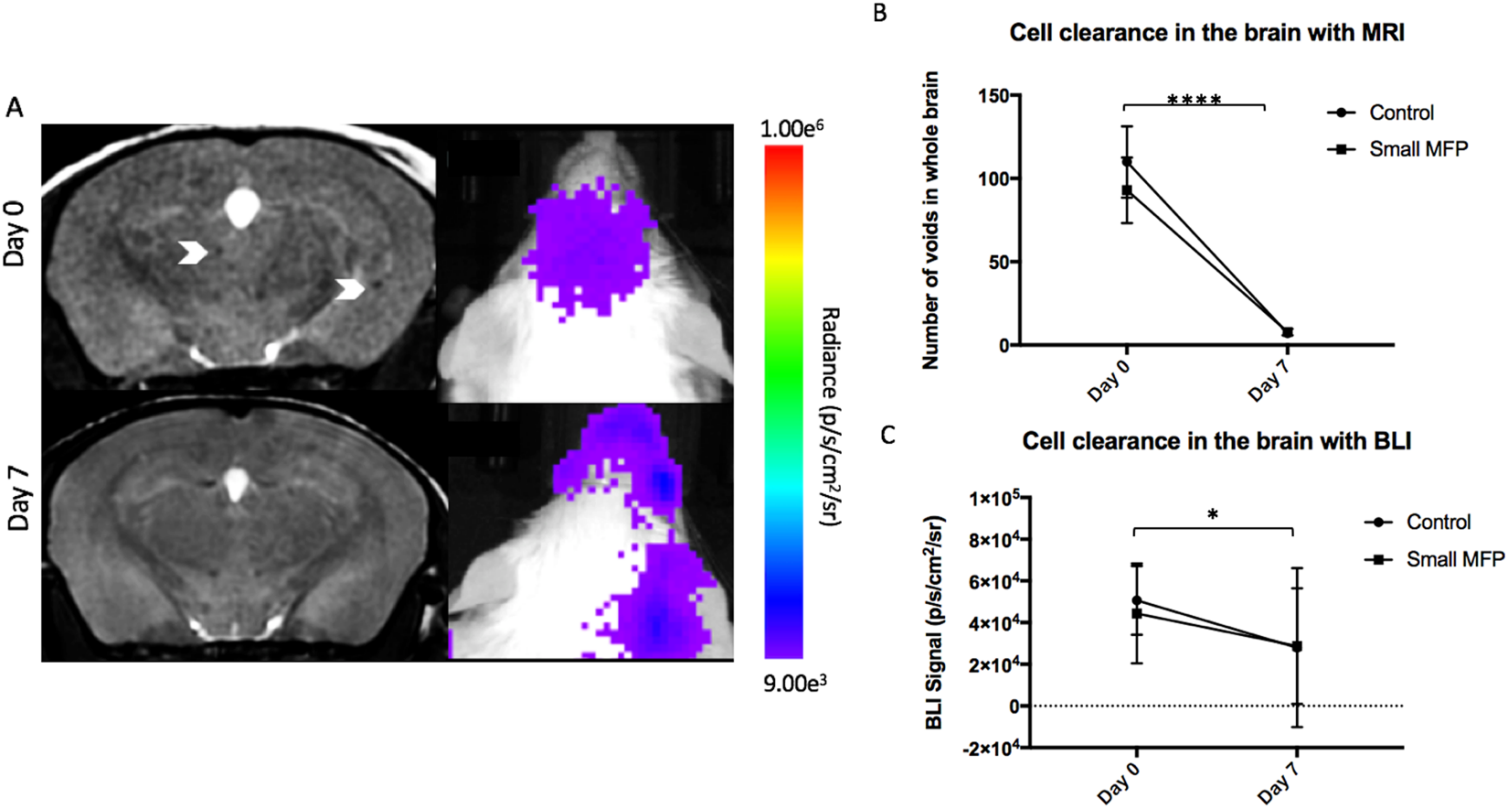
(**A**) BLI and MR imaging data illustrates the signal loss over time with cell clearance in the brain of small MFP and control mice. (**B**) The number of signal voids in the brain decreases from days 0 to 7 as cancer cells are cleared from the brain. (**C**) BLI signal also decreases from day 0 to 7 as cancer cells are cleared from the brain. Data is presented as mean +/− SD. *Indicates p < 0.05.

For mice in the small MFP experiment we looked at imaging data over time and Fig. 2 illustrates the MRI and BLI signal loss over time representing viable cancer cell clearance in the brain for BLI as well as potential iron label dilution in MR images. The number of signal voids in the brain significantly decreased from day 0 (109.9 ± 7.58 voids) to 7 (7 ± 0.27 voids) for control mice and from day 0 (92.88 ± 6.96) to 7 (7.75 ± 0.70; p < 0.0001; Fig. 2B) for small MFP mice. BLI signal in the brain also significantly decreased for control mice from day 0 (5.1 × 10^4^ ± 5.8 × 10^3^ p/s/mm^2^/sr) to 7 (1.5 × 10^4^ ± 3.6 × 10^3^ p/s/mm^2^/sr) and for small MFP mice from day 0 (4.4 × 10^5^ ± 8.5 × 10^3^) to day 7 (2.9 × 10^5^ ± 9.8 × 10^3^; p < 0.01; Fig. 2C).

**Figure 3:**
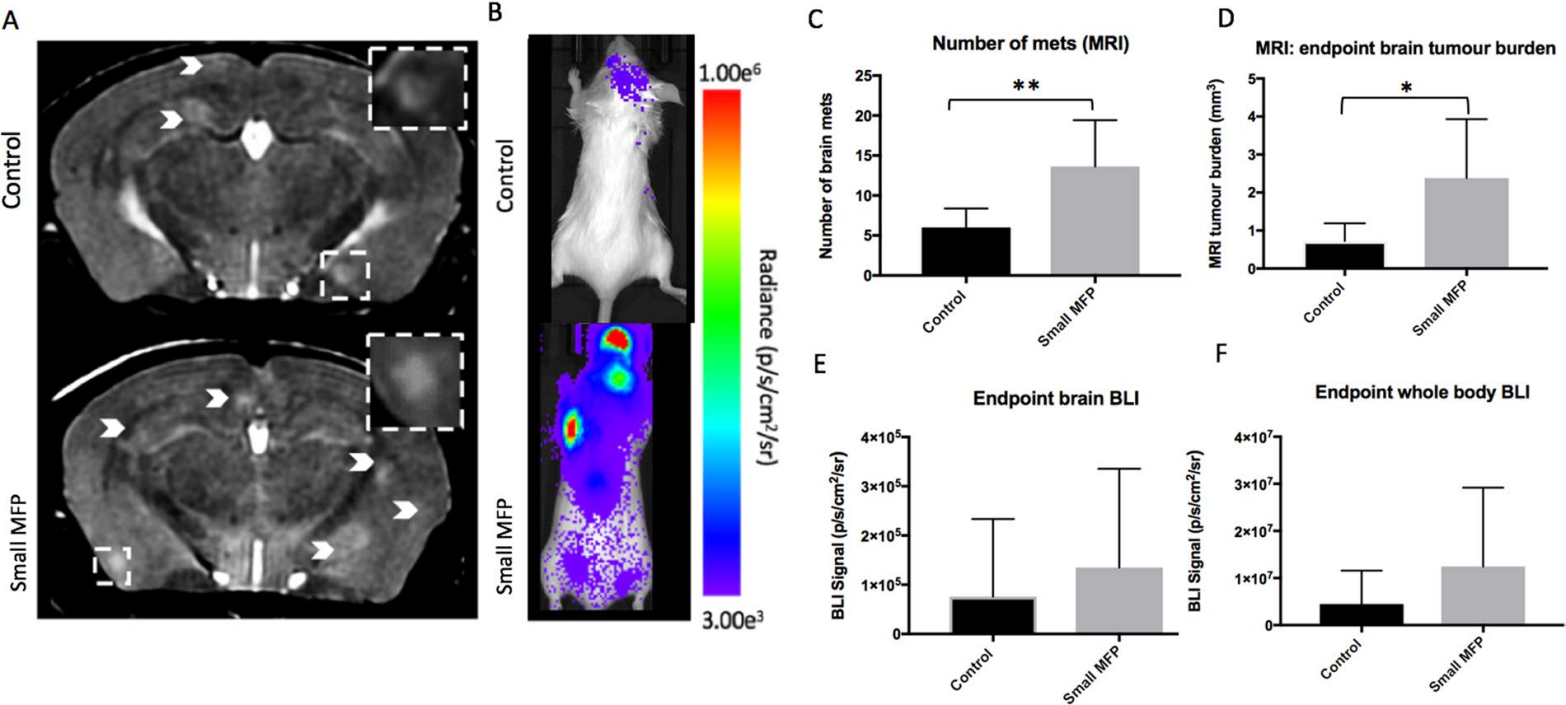
(**A**) At day 14, brain metastases appeared as regions of hyperintensity by MRI, and (**B**) regions of BLI signal in the brain and body. (**C**) Mice with a primary small MFP tumor had significantly more brain metastases than mice without a primary tumor. (**D**) Mice with a primary tumor had significantly more total brain tumor burden than mice without a primary small MFP tumor. (**E**,**F**) There were no significant differences in BLI signal in the brain or body between mice with and without a primary small MFP tumour. Data is presented as mean +/− SD. *indicates p < 0.05; **Indicates p < 0.01.

**Figure 4:**
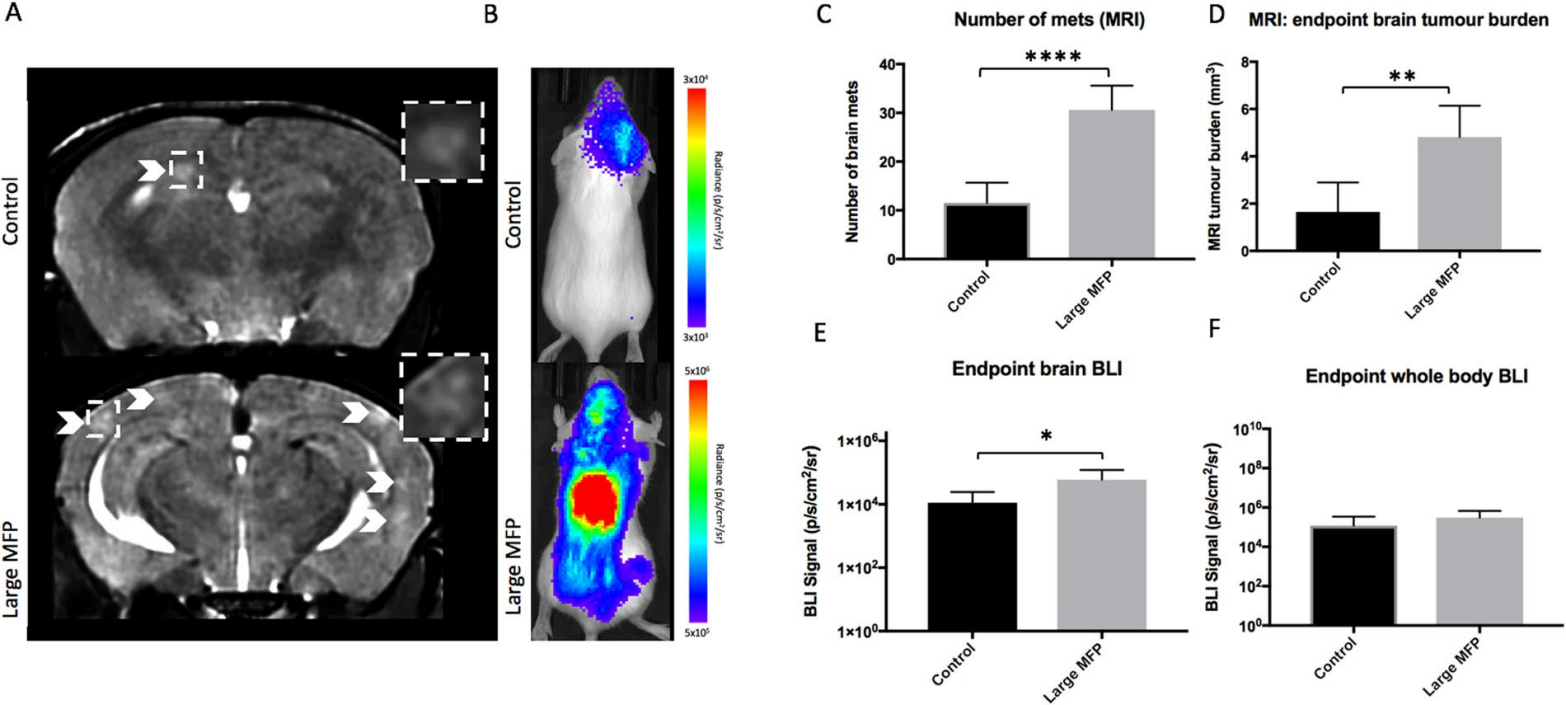
(**A**) At day 14, brain metastases appeared as regions of hyperintensity by MRI, and (**B**) regions of BLI signal in the brain and body. (**C**) Mice with a large MFP tumor had significantly more brain metastases than mice without a primary tumor. (**D**) Mice with a large MFP tumour had significantly more total brain tumor burden than mice without a primary tumor. (**E**,**F**) Similarly, mice with a large MFP tumour had significantly more BLI signal in both the brain and the body compared to control mice. Data is presented as mean +/− SD. *Indicates p < 0.05; **indicates p < 0.01.

At day 14, brain metastases appeared as regions of hyperintensity by MRI in all twenty-nine mice that made it to endpoint as well as two large MFP mice that had an early endpoint of day 10 due to illness (Fig. 3 - Small MFP group; Fig. 4 – Large MFP group). An additional mouse in the large MFP group had to be sacrificed at day 9 due to signs of illness but endpoint MRI was not performed and thus this mouse was not included in our endpoint analysis. Figures 3A and 4A show an MR slice from a representative mouse from each group. MR image analysis revealed that mice with a small primary MFP tumour (13.63 ± 2.05 tumours) had significantly more brain metastases than control mice (6 ± 0.84 tumours) (Fig. 3C). We also found that mice with a small MFP tumour (2.38 ± 0.55 mm^3^) had significantly higher total brain tumour volume than control mice (0.72 ± 0.17 mm^3^) (Fig. 3D). Similarly, we found that mice with a large primary MFP tumour (30 ± 2.22 tumours) had significantly more brain metastases than control mice (12 ± 1.48 tumours) (Fig. 4C). We also found that mice with a large MFP tumour (4.81 ± 0.59 mm^3^) had significantly increased total brain tumour volume than control mice (1.65 ± 0.44 mm^3^) (Fig. 4D).

Figure 3B/4B show BLI images of a representative mouse from each group at endpoint (day 14). All 13 MFP mice that made it to endpoint had BLI signal in the brain while only 13 of 16 control mice had signal in the brain. An additional 2 MFP mice (large) had BLI signal in the brain at day 10 (day of sacrifice) which were included in our endpoint measurements of brain tumour burden. BLI signal in the brain at endpoint was not significantly different between mice with (1.1 × 10^5^ ± 5.1 × 10^4^ p/s/mm^2^/sr) and without (4.3 × 10^4^ ± 3.5 × 10^5^ p/s/mm^2^/sr) a small MFP tumour (Fig. 3E). However, BLI signal in the brain at endpoint was significantly different between mice with (5.9 × 10^4^ ± 2.2 × 10^4^ p/s/mm^2^/sr) and without (1.1 × 10^4^ ± 4.7 × 10^3^ p/s/mm^2^/sr) a large MFP tumour (Fig. 4E). All MFP and control mice had BLI signal in the body at endpoint. Similarly, whole body BLI signal was not significantly different between mice with (1.2 × 10^7^ ± 5.9 × 10^6^ p/s/mm^2^/sr) and without (4.5 × 10^6^ ± 2.5 × 10^6^ p/s/mm^2^/sr) a primary MFP tumour (Fig. 3F) or between mice with (3.1 × 10^5^ ± 1.7 × 10^5^ p/s/mm^2^/sr) and without (1.2 × 10^5^ ± 7.9 × 10^4^ p/s/mm^2^/sr) a large MFP tumour (Fig. 4F).

### Histology and whole-mouse cryo-fluorescence imaging

**Figure 5:**
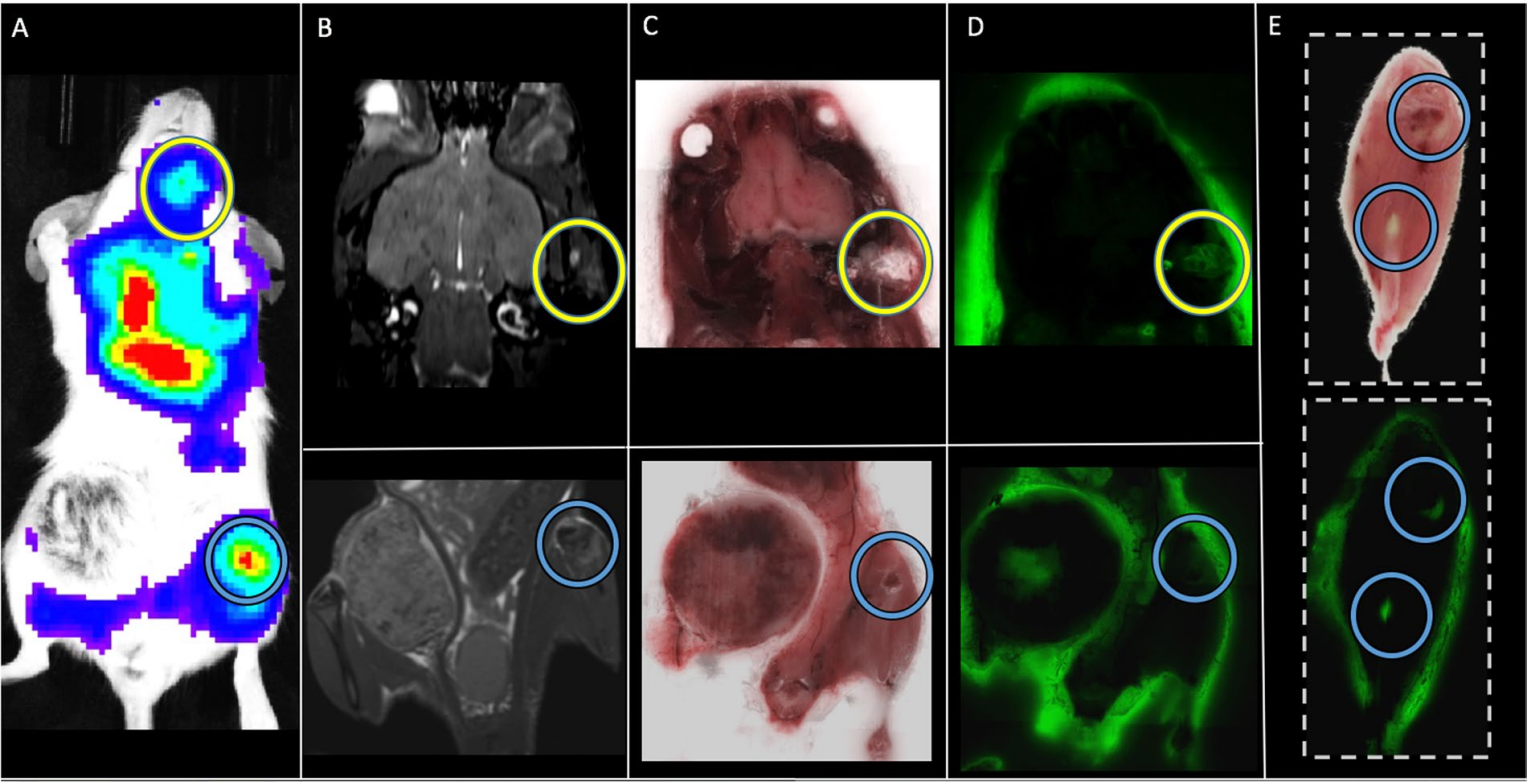
In a mouse from the large MFP group we were able to detect distant metastases (head circled in yellow; bone circled in blue) with BLI (**A**) and match them to corresponding MR (**B**) and brightfield (**C**,**E**) and fluorescence (**D**,**E**) cryoviz images.

**Figure 6:**
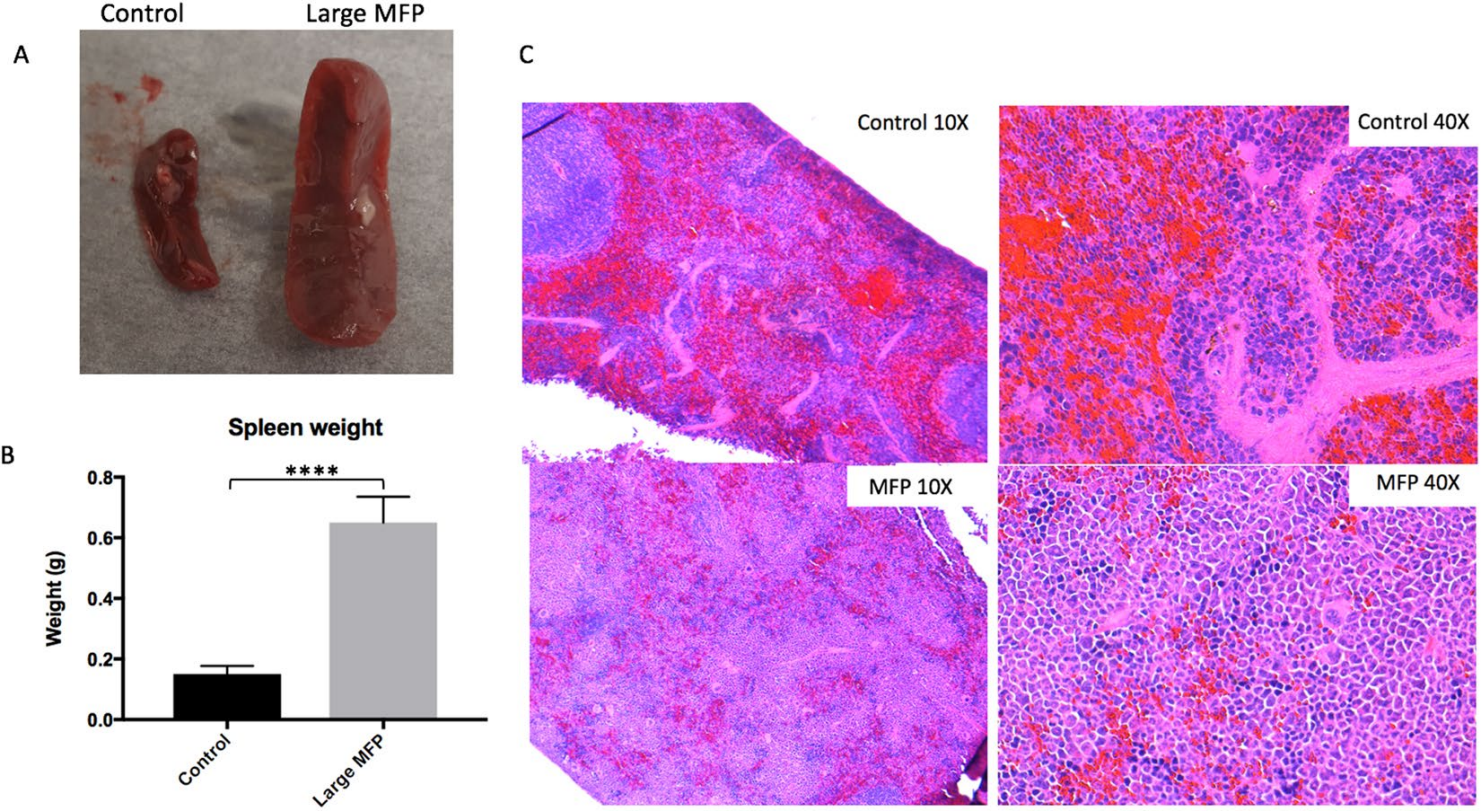
(**A**,**B**) Spleens from large MFP tumor-bearing mice were enlarged and significantly higher in weight(g) than spleens collected from control mice. (**C**) Normal spleen histology was observed in control mice (Top) but it was found that the spleens isolated from large MFP mice showed a reduction in red pulp (bottom) compared to control mice and normal spleen histology. Data is presented as mean +/− SD. ****Indicates p < 0.0001.

In one large MFP mouse, lung metastases were detectable in whole-body MR images but not in the respective control mouse (Suppl. 3). In one small MFP mouse the presence of tumours was also confirmed in *ex vivo* BLI (Suppl. 4). Coronal MR images show corresponding orientation to BLI signal; *in vivo* and *ex vivo* whole body BLI were also matched presenting signal in the abdominal region. Fluorescence microscopy demonstrated that the location of 4T1BR5-Red-FLuc/GFP cells corresponded well with H&E as well as hyperintense signal representing tumors in the corresponding MR slice (Suppl. 5). This confirms that metastases detected with MRI contain 4T1BR5-Red-FLuc/GFP cells. In two mice, the presence of tumors was also confirmed using cryo-fluorescence imaging which has the ability to perform whole-body brightfield and fluorescence imaging throughout the entire mouse. Cryo-fluorescence imaging allowed for the detection of metastases at day 14 in both the bone and other areas of the body (Fig. 5). We were able to match some of the metastases seen with cryo-imaging to MR tumours. Cryo-fluorescence imaging also allowed for the localization of secondary metastases seen with BLI. Due to light scattering and the depth limitation of BLI, we cannot be certain where within the body as well as the number of metastases the signal is coming from; cryo-imaging allows us to further explore a region of interest and find out exactly where the tumor is located. Finally, we found that spleens from large MFP tumour-bearing mice (0.65 ± 0.03 g) were enlarged with significantly higher weights compared to spleens collected from control mice (0.15 ± 0.01 g) (p < 0.0001; Fig. 6A,B). Normal spleen histology was observed in control mice but it was found that the spleens isolated from large MFP mice showed a reduction in red pulp compared to control mice (Fig. 6C).

## Discussion

Clinical and experimental evidence suggests that a primary tumour can both accelerate (CTE) or retard (CTR) the growth of distant metastases^4–14^. In this study, we found CTE effects in the syngeneic 4T1 model using multimodality imaging. The presence of a primary MFP tumour increased the number and total volume of brain metastases as measured with MRI and BLI (in the large MFP group). Secondly, this effect was amplified when the primary tumour was larger at the time of secondary injection of experimental metastatic cells. To our knowledge, this is the first report of multimodality imaging being used to study the impact of a primary tumour on metastatic outgrowth in the 4T1 model.

In our previous studies in immune compromised mice (nu/nu) we found the presence of a human primary breast tumour (MDA-MB-231) significantly inhibited the growth of MDA-MB-231BR brain metastases (i.e., a CTR effect)^16^. For this study, MRI revealed fewer brain metastases developed in mice with than without a primary tumour. Interestingly, using cellular MRI, we also found that significantly more signal voids (representing non-dividing cancer cells) persisted in endpoint images of mice that had a primary tumour compared to mice that did not. This highlighted that a primary tumour can in part maintain cancer cell dormancy and mitigate overt metastases formation, a potential new mechanism for CTR. We could not assess endpoint signal void number in the current study due to the very low number of cells arresting and remaining in the brain at endpoint. In future studies, we could shorten the experimental timeline to enable a greater number of cells injected into the heart, which in turn could result in more voids persisting at endpoint.

Our results between the two models suggests the immune system may play a role in mediating CTR or CTE effects, as also suggested previously by others. Janik *et al*., found that immune competent mice are first protected and then made more susceptible to the growth of lung metastases by the presence of a progressively growing primary tumour^7^. Similarly, Vaage *et al*., showed that immunity decreases around day 20 after implanting tumour cells into mice and that this phenomenon was associated with an excess of tumour antigen, and that after removal of the primary tumour immunity is quickly restored^25^. This may provide support as to why we found an amplified CTE effect in our large MFP mice. This cohort of mice had 14 days of primary tumour growth (compared to 7 days in small MFP mice) before secondary injection, followed by 14 days of metastatic growth. This theoretical 20-day period would fall fairly early (6 days) into the period of metastatic growth, leaving these mice vulnerable to enhanced metastatic growth due to decreased immunity. Whether the immune system is playing a significant role in the CTE effects seen in this study could be determined by moving our 4T1/4T1BR5 model from BALB/c mice into an immune compromised mouse (e.g., nu/nu), expecting either a CTR effect or a lesser CTE effect than seen in the present study. Similarly, we may see a more significant CTR effect by studying the MDA-MB-231/MDA-MB231BR model into more immune deficient mice than used before (nu/nu) such as nod-scid-gamma (NSG) mice, or evaluating whether a CTE effect might appear in humanized mice.

An immunological component to the CTE effects seen is also suggested from our findings in the spleens from our animals. We found splenomegaly in tumour-bearing mice however, this finding was associated with a reduction in red pulp and not differences in white pulp. Kirstein *et al*., found splenomegaly in tumour-bearing animals was associated with tri-lineage extramedullary hematopoiesis as well as a reduction in white pulp in their mouse model of CTR^14^. Other groups have also seen splenomegaly in tumour-bearing animals, specifically in the 4T1-model. Thus, differences in the ratio of red vs. white pulp in splenic tissue needs to be studied further to determine whether this is an effect of prolonged 4T1 tumor growth or an effect of CTE.

Past studies have shown that the size of the primary tumour plays a key role in whether a CTE or CTR effect is observed. For instance, Bruzzo *et al*., previously demonstrated secondary tumour growth can be either stimulated or inhibited depending on the ratio between the mass of the primary tumour relative to that of the secondary tumour implant. They found that high ratios tended to cause inhibition of secondary tumour growth while low ratios induced a stimulation effect^8^. For the present study, we injected our secondary cancer cell line at an early time point (7 days post MFP injection) when the primary MFP tumour is relatively small. We did this in an effort to best match the size of our MDA-MB-231 model at the time of secondary injection. However, despite similar primary tumour size at the time of secondary injection, these two models produced opposite effects (CTE/CTR). This may be due to differences in cell line aggressiveness; 4T1 metastases can be detected as early as 7 days whereas the less aggressive MDA-MB-231 model may take between 2–3 weeks before metastases start to form in the brain. This is significantly more time for the MDA-MB-231 primary tumour to grow (increasing the ratio between primary and secondary) to produce a CTR effect on secondary tumour growth. In the 4T1 model, as metastases quickly develop, the ratio between primary and secondary tumours becomes smaller over time. In the current study, we also injected a second cohort of animals at a late time point (14 days post MFP injection) when the primary tumour is relatively large to try to increase the ratio between primary and secondary tumour (producing a CTR effect). However, we found that increasing the size of the primary tumour at the time of secondary injection significantly amplified our CTE effect.

An important step in the metastatic cascade is the initial arrest of cancer cells. When cancer cells enter the circulation, they can travel through the bloodstream and arrest in the capillary beds of distant sites throughout the body and extrasvasate into the interstitial space where they can begin to form new tumours. For breast cancer patients, metastases are most commonly found in the brain, bone, lung and liver^26^. Our multimodality imaging tools allow us to noninvasively monitor both the number (MRI) and viability (BLI) of arrested single cancer cells in the brain, allowing us to for the first time study the effect of a primary tumour on cancer cell arrest as well as cancer cell clearance. In the present study, we found there was not a significant difference in viable cancer cell arrest in the brain between mice with a primary MFP tumour and control mice. We also found that there was not a significant difference in cancer cell clearance from the brain, suggesting the presence of a primary MFP tumour does not influence the number of viable, iron labeled cancer cells that arrest in the brain at day 0, nor does it affect the clearance of these cells, indicating that in this model the increased tumour growth we detected in mice with primary tumors at endpoint is not due to effects at the early stages of the metastatic cascade. Another important step is the intravasation of cells from the primary tumour. We will explore this in the future by evaluating the effects of removal of the primary tumour on spontaneous metastasis formation.

BLI was performed at endpoint on all mice to measure viable tumour burden in both the brain as well as whole body. Although our MR analysis shows a strong CTE effect in the brain of both the small and large MFP mice, we only found significant differences in brain bioluminescence signal between mice with a large primary MFP tumour and control mice. Previous studies have also shown disagreement in tumour volume and BLI measurements at endpoint^27,28^. Although surprising, it should be noted that MRI and BLI are measuring two different tumour characteristics; MRI is measuring tumour volume, which is affected by many things including the number of tumour or stromal cells present and the presence of edema, amongst other things, whereas BLI is providing a measure of the viability of the engineered cancer cells over time. Moreover, BLI is not without its caveats, particularly when studying metastatic disease. First, BLI signal is depth dependent. Small shallow tumours will appear brighter or equivalent in signal to larger deep tumours. Hence, BLI should be used for monitoring relative tumour viability over time rather than considering BLI signal at any one-time point to represent a direct measure of absolute tumour burden. Second, necrosis may be present within some of the metastases. While our MRI tumour burden may be composed of both live and dead tumour tissue, BLI signal is representative of viable cancer cells only. Third, any areas of hypoxia in a tumour may demonstrate lower BLI signal due to the need for oxygen in the luciferase/luciferin reaction. Despite these caveats, our previous work has shown that the information provided by both imaging modalities over time makes them very complementary technologies for studying cancer metastasis^18^.

To account for the possibility of spontaneous metastases developing from our naïve primary cell line and contributing to the main findings in this study, our metastatic cell line was engineered to express GFP. As a result, we can confirm that any GFP-positive metastases came from our intracardiac injection of metastatic cells, whereas GFP-negative metastases are likely spontaneous metastases. In our endpoint histology, we did not find any GFP-negative metastases (all 19 tumours found were GFP-positive), suggesting that all brain metastases detected with MRI at endpoint came from our metastatic cell line and not the primary tumour. In addition, we performed a study where we injected 300,000 4T1-FLuc-GFP cells into the mammary fat pad of immune competent mice. We then monitored primary tumour growth over 28 days with BLI. In this 4-week period we were not able to detect distant metastases in the brain or any other organ. Since BLI is more sensitive than MRI, we can be fairly confident that there are also not any MR-detectable metastases at this time point. This has also been shown by other groups studying spontaneous metastasis of 4T1 cells with BLI^29^. For example, Tao *et al*., shows that by week 6 after mammary fat pad injection of 1 × 10^6^ 4T1 cells, only 1 of 6 mice had BLI-detectable metastases in the brain. This is more than three times the number of cells that we are injecting for the current study and thus, we do not expect spontaneous metastases to form in our 4-week time line. In this study, we also show significant differences in endpoint tumour burden in the brain with BLI measures. If a large portion of the MRI tumour burden was from spontaneous metastases, we predict we would not have seen the differences we found in our BLI measurements.

In this work, we applied cellular and molecular imaging tools to evaluate the effect of a primary breast tumour and its size on the growth of brain metastases in the immune competent 4T1 mouse model. We found total brain tumour burden was significantly greater in mice with a primary MFP tumour (Small or Large) compared to those without. We also found that mice with a large MFP tumour had significantly more BLI signal in the brain at endpoint compared to control mice. Interestingly, using *in vivo* BLI and MRI we could determine that these differences in endpoint tumour burden were not related to differences in the initial arrest or clearance of viable cells in the brain, which suggests that the presence of a primary tumour can increase the proliferative growth of brain metastases in this syngeneic 4T1 mouse model. Future work will utilize our imaging tools to explore the role of the immune system in promoting or preventing metastatic growth. Understanding the different immunological and/or molecular mechanisms of stimulation (CTE) versus inhibition (CTR) will be extremely valuable in finding new therapeutic options for breast cancer patients.

## Electronic supplementary material

Supplementary Information
