# Supplementary material for: Multimodality cellular and molecular imaging of concomitant tumour enhancement in a syngeneic mouse model of breast cancer metastasis
Source: Sci Rep. 2018 Jun 12;8:8930. doi: 10.1038/s41598-018-27208-4 (PMC5997674; doi:10.1038/s41598-018-27208-4)
Supplement: Supplementary file 1 — Supplementary Information [file 41598_2018_27208_MOESM1_ESM.pdf]

Multimodality cellular and molecular imaging of concomitant tumour enhancement in a syngeneic mouse model of breast cancer metastasis

Katie M. Parkins[1,2], Veronica P. Dubois [1,2], Amanda M. Hamilton[1], Ashley V. Makela[1,2], John A. Ronald[1,2,3], Paula J. Foster*[1,2]

[1]Robarts Research Institute, The University of Western Ontario, London, Ontario, Canada
[2]The Department of Medical Biophysics, The University of Western Ontario, London, Ontario, Canada
[3]Lawson Health Research Institute, London, Ontario, Canada

Corresponding Author: pfoster@robarts.ca
Imaging Research Laboratories, Robarts Research Institute
1151 Richmond Street North London, Ontario, Canada N6A 5B7
Phone:(519) 931-5777 Ext. 24316

Supplementary Information File

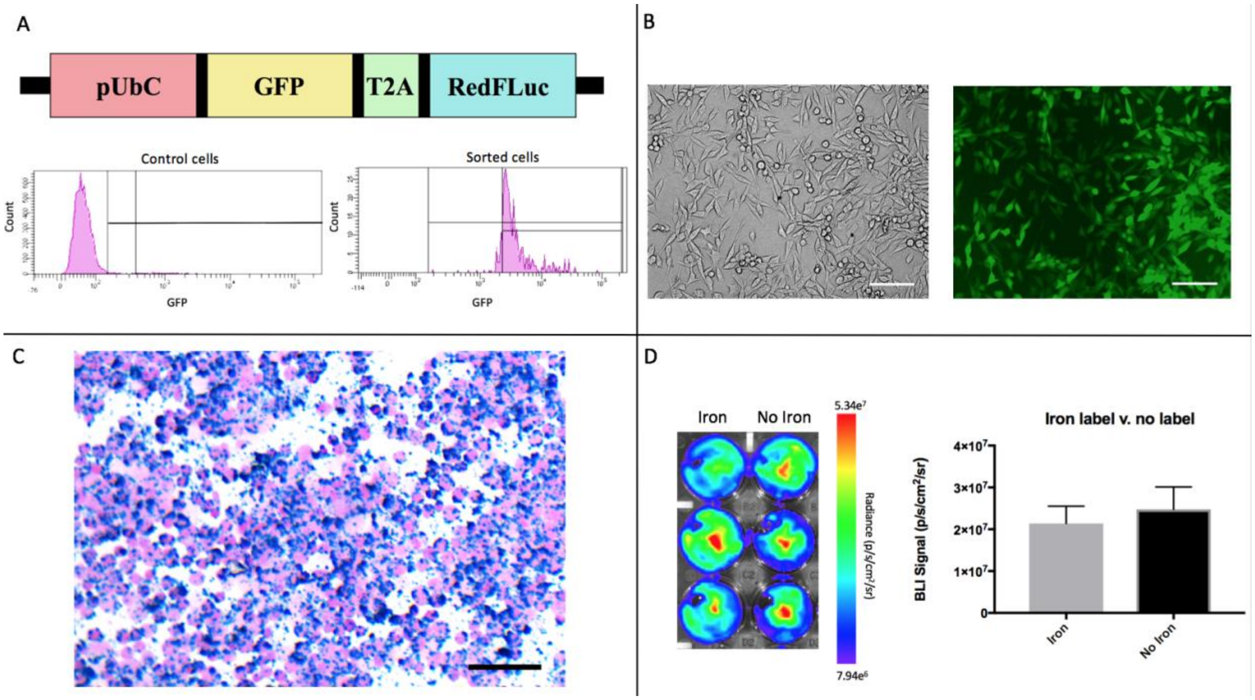

Suppl. Figure 1: A/B) 4T1-BR5 cells were transduced and sorted to stably co-express Red-FLuc/GFP using a commercial lentiviral vector (Scale bars = 100 microns). **C)** The resultant 4T1BR5-Red-FLuc/GFP cells were efficiently labeled with > 90% of cells labeled with MPIO prior to intracardiac injection (Scale bar = 200 microns). **D)** No significant difference in BLI signal was detected in 4T1BR5-Red-FLuc/GFP cells that were labeled with MPIO and cells that were not labeled. Data is presented as mean +/- SD.

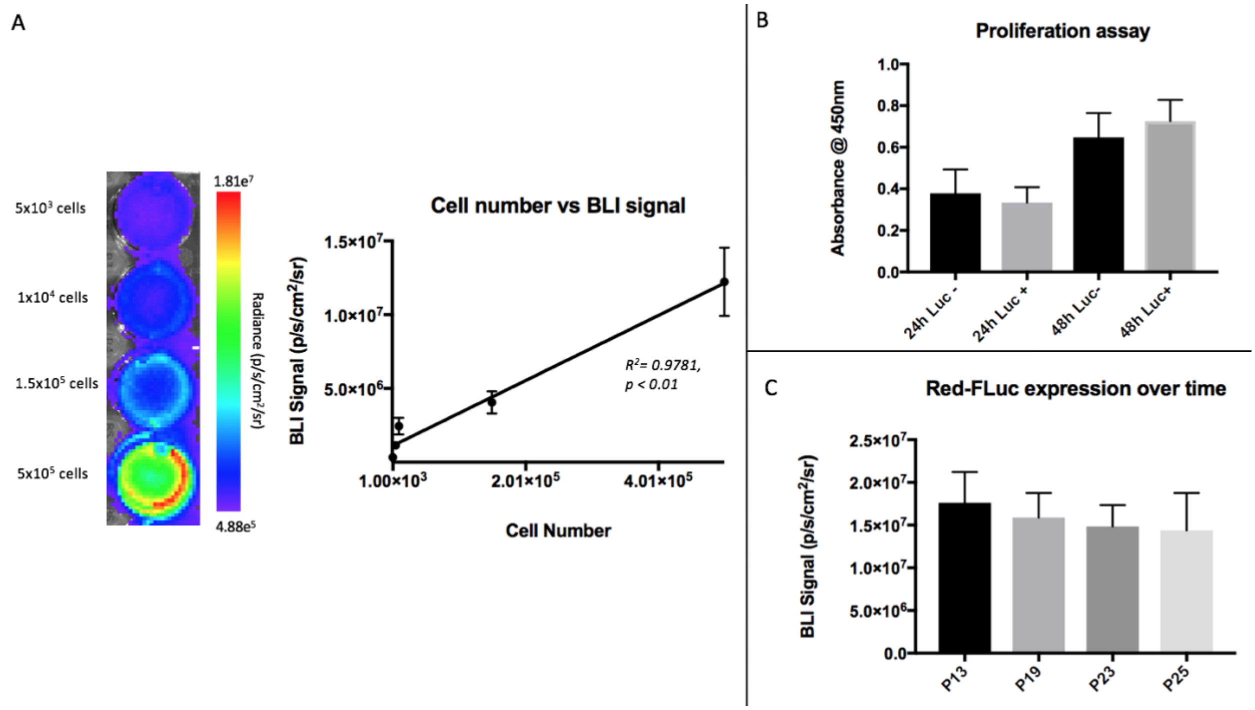

Suppl. Figure 2: A significant positive correlation was found between the number of 4T1BR5-Red-FLuc/GFP cells and BLI signal (A). There was not a significant difference in cellular proliferation detected between naïve 4T1-BR5 and 4T1BR5-Red-FLuc/GFP cells (B). 4T1-BR5-Red-Fluc/GFP cells showed no significant change in Red-Fluc expression over multiple passages in vitro (C).

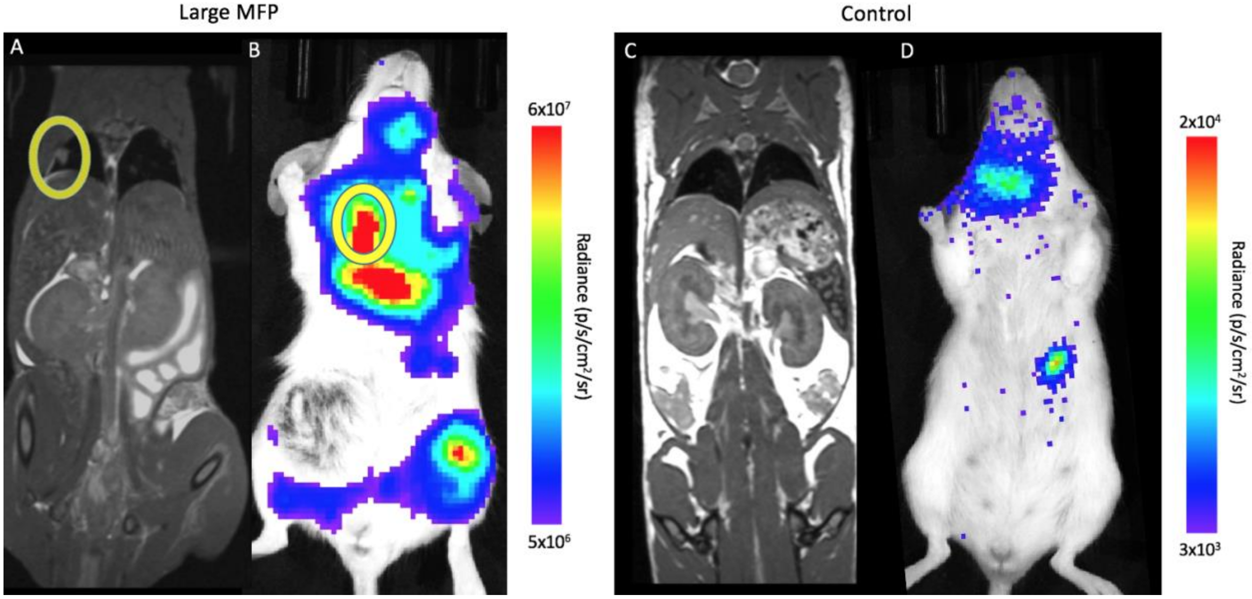

Suppl. Figure 3: A) Lung metastases (circled in yellow) were detectable at day 14 with whole body MRI in mice with a large MFP primary tumor, but **C)** not control mice. **B)** BLI signal was also detected in the lung region of large MFP mice on day 14, but **D)** not control mice.

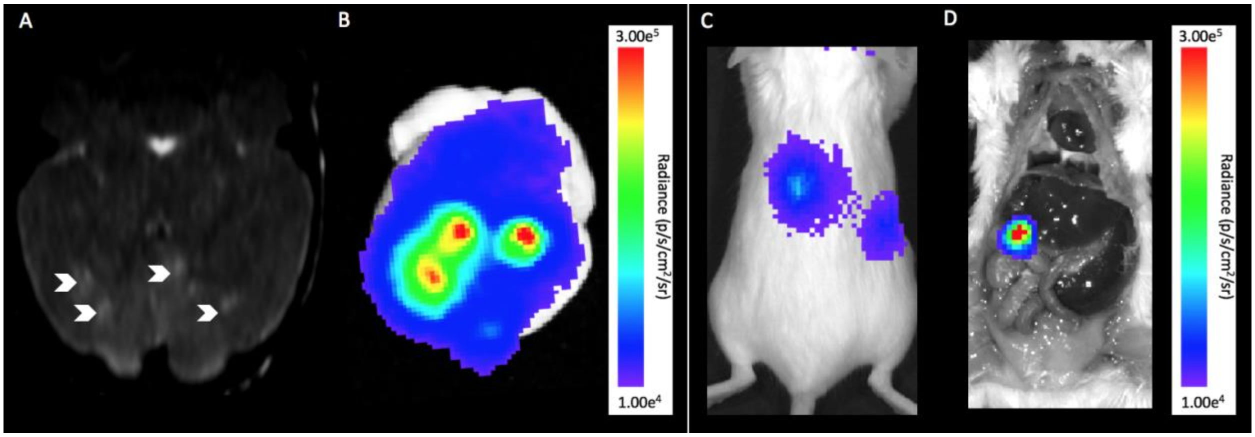

Suppl. Figure 4: A representative in vivo MR image of the brain (A) and corresponding ex vivo BLI (B); *in vivo (C)* and *ex vivo (D)* whole body BLI were also matched presenting signal in the abdominal region.

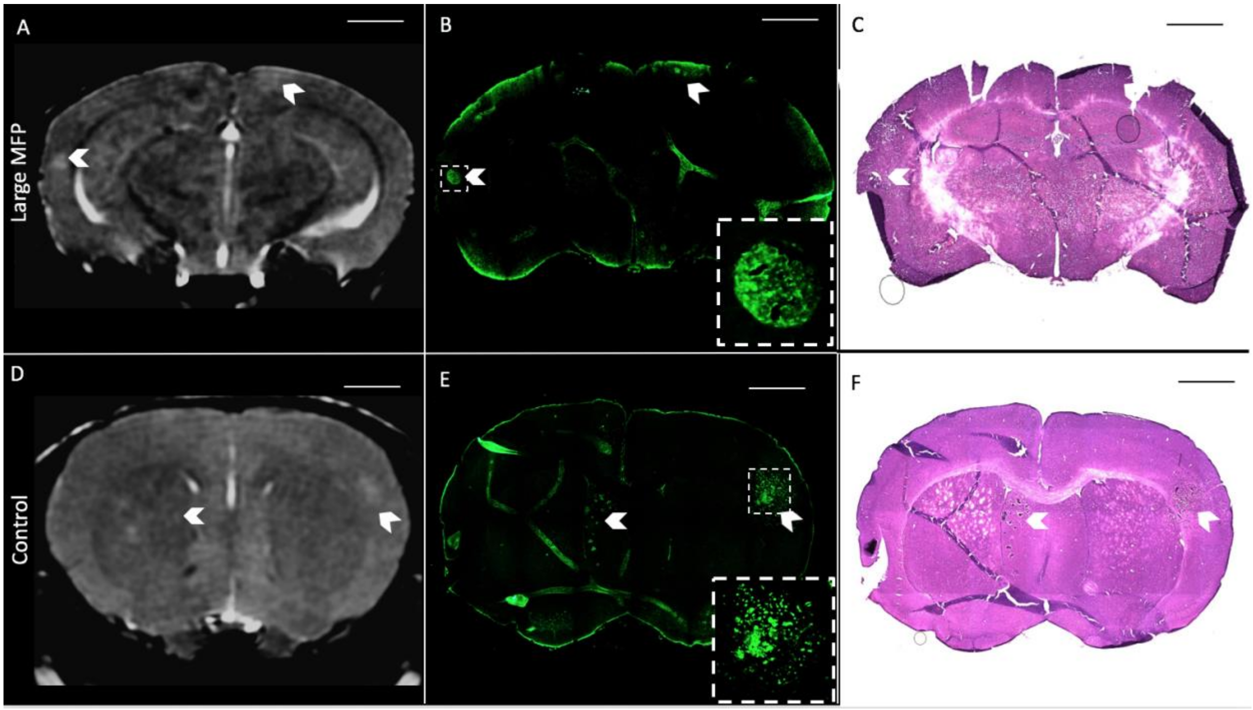

Suppl. Figure 5: A representative MR slice **(A/D)** corresponded well with GFP positive cells **(B/E)** and H&E staining **(C/F)** confirming that MR-detectable metastases contain 4T1BR5-Red-FLuc/GFP cells. Scale bars = 500 microns.
